# Environmental Stability and Residual Stresses in Zirconia Femoral Head for Total Hip Arthroplasty: *In Vitro* Aging versus Retrieval Studies

**DOI:** 10.1155/2015/638502

**Published:** 2015-06-03

**Authors:** Masanori Arita, Yasuhito Takahashi, Giuseppe Pezzotti, Takaaki Shishido, Toshinori Masaoka, Keiji Sano, Kengo Yamamoto

**Affiliations:** ^1^Department of Orthopaedic Surgery, Tokyo Medical University, 6-7-1 Nishishinjuku, Shinjuku-ku, Tokyo 160-0023, Japan; ^2^Department of Bone and Joint Biomaterial Research, Tokyo Medical University, 6-7-1 Nishishinjuku, Shinjuku-ku, Tokyo 160-0023, Japan; ^3^Ceramic Physics Laboratory, Kyoto Institute of Technology, Sakyo-ku, Matsugasaki, Kyoto 606-8585, Japan

## Abstract

The objective of this study was to compare the low temperature degradation (LTD) behavior of femoral heads made of 3Y-TZP as observed on retrievals with that induced *in vitro* upon prolonged exposures to a hydrothermal environment. The time-dependent evolution of tetragonal-to-monoclinic transformation and the related residual stresses were nondestructively monitored by Raman microspectroscopy. An increasing intensification of tensile and compressive stresses was detected with increasing hydrothermal aging duration in tetragonal and monoclinic phases, respectively. The dependence of monoclinic fraction upon exposure time was rationalized through the Mehl-Avrami-Johnson (MAJ) formalism in order to interpret the LTD process according to a two-step mechanism of formation and growth of monoclinic nuclei. *In vitro* results were compared to *in vivo* monoclinic contents in the same type of 3Y-TZP head retrievals after implantation periods of 1.6–16.6 y, also including literature data previously reported by other authors. One-hour exposure under the selected aging condition is estimated to correspond to *in vivo* exposures of 4 and 2 years according to ISO and ASTM criteria, respectively. A critical review of these two criteria according to the present analyses revealed that the ASTM simulation predicts more closely the *in vivo* results as compared to the ISO one.

## 1. Introduction

Zirconia (ZrO_2_) bearings have been considered as a valid alternative to alumina bearings in total joint replacement (TJR). The most attractive characteristics of ZrO_2_ are its excellent flexural strength and fracture toughness, which are significantly higher than those of alumina (Al_2_O_3_) [1]. So far, the ZrO_2_ material used in orthopedics has predominantly consisted of a partially stabilized tetragonal phase with a content of 3 mol% of yttria (Y_2_O_3_), which is typically referred to as 3Y-TZP (i.e., 3 mol% Y_2_O_3_-stabilized tetragonal ZrO_2_ polycrystal) [1–3]. Unlike metallic joint implants made of cobalt-chrome or stainless steel [4], oxide ceramics are supposed to be more stable in biological environment since, in principle, they possess less driving force for structural degradation in air. Nevertheless, it has been well recognized that, in human body, the 3Y-TZP material is metastable and can transform from the tetragonal to the monoclinic (*t* → *m*) polymorph under the combined effects of biological (aqueous) and mechanical stress environments (e.g., frictional wear, body weight, and impingement). When acting against advancing cracks, this phenomenon plays a significant role in increasing fracture toughness by generating a strong crack-shielding effect as a consequence of 3~4% volume expansion (i.e., a mechanism referred to as transformation toughening) [1–3, 5]. However, if the *t* → *m* transformation becomes environmentally driven and uncontrollably occurs prior to crack propagation, the material undergoes a significant loss of crack growth resistance, strength, and reliability [1–3, 5]. This latter mechanism is usually referred to as low temperature degradation (LTD) and has been regarded as the “Achilles' heel” of 3Y-TZP when employed in load-bearing applications, leading to catastrophic fracture [1, 2, 6] and surface roughening [2, 3, 7]. Since the above two mechanisms occur competitively during* in vivo* implantation, the time-dependent LTD phenomenon makes orthopedic surgeons hesitant about using ZrO_2_ at the time of arthroplastic intervention.

In the above contexts, the stability of the tetragonal phase can largely affect the clinical outcome and long-term survivorship of joint implants made of 3Y-TZP prosthesis. Thus, it is of great importance to experimentally simulate the time-dependence of LTD behavior of 3Y-TZP and the related changes in residual stress fields at its bearing surface, which could provide a reasonable prediction of* in-vivo* performance and durability. The aim of this study is to estimate to which extent an* in vitro* aging test, standardized for biomedical grade ZrO_2_, could be valid for predicting the* in vivo* resistance to LTD. For this purpose, we artificially accelerated the LTD phenomenon in commercially available 3Y-TZP femoral heads by means of a prolonged exposure to water vapor atmosphere (134°C, 2 bars of pressure) on the basis of ISO and ASTM testing conditions [8–11]. The time-dependent degradation of 3Y-TZP heads was assessed by means of confocal Raman microprobe spectroscopy in terms of increases in monoclinic volume fraction as well as in surface residual stress magnitude. In order to screen nondestructively the aging behaviors, the spectroscopic evaluations were made in confocal mode from the articular surface down to subsurface regions of the femoral heads. Furthermore, the LTD kinetics of the* in vitro* aged heads was compared with that obtained from head retrievals of the same brand subjected to long-term implantation* in vivo* (>15 years). Comparisons were also made with the surface monoclinic fractions analyzed previously by other authors for short- and middle-term retrievals (1.6~11 years) via X-ray diffraction (XRD) analysis [12–14].

## 2. Materials and Methods

### 2.1. Unused 3Y-TZP Femoral Heads and* In Vitro* Aging Test

The 28 mm sized 3Y-TZP femoral heads (*n* = 3) were provided by Biomet Inc. (Warsaw, IN). These were manufactured by Saint-Gobain Desmarquest Inc. (Evreux, France). These heads were distributed with the trade name of Prozyr (currently out of market since 2001). All the Prozyr heads used in this study were produced by batch-furnace sintering, and not by tunnel furnace sintering which historically had resulted in the unprecedented number of reports of fractured Prozyr heads [2, 6]. The head samples were subjected to no further washing or manipulation prior to* in vitro* aging test and spectroscopic characterizations. The average grain size of the heads was 0.5 *μ*m. In this paper, the as-received samples are simply referred to as “Head (I)” (Table 1). The accelerated aging test was performed at 134°C under 2 bars water steam, namely, the conditions that satisfy both ISO and ASTM standards [8–11]. The aging test was carried out at 2.5 h intervals up to 20 h using a high-pressure steam sterilizer (TOMY SX-300, Tomy Seiko, Co., Tokyo, Japan). After sequential intervals of 2.5 h in autoclave, the monoclinic content and the residual stress field were monitored using confocal Raman microprobe spectroscopy (cf. forthcoming Section 2.3).

### 2.2. Retrievals of 3Y-TZP Femoral Heads and Their Clinical Background

In order to compare the* in vitro* LTD behavior of the pristine Head (I) with the actual* in vivo* performance of the same material, two Prozyr femoral head retrievals were also investigated by Raman microprobe spectroscopy. These retrievals were implanted in female patients for 15.1 and 16.6 years (i.e., henceforth referred to as Heads (II) and (III), resp.). Clinical information is summarized for both heads in Table 1. The diameter of Heads (II) and (III) was 28 mm. Both heads articulated against acetabular liners made of conventional (noncrosslinked) ultra-high molecular weight polyethylene (ArCom, Biomet Inc., Warsaw, IN) with the outer diameter of 48 mm. The causes of revision surgery were infection (Head (II)) and liner wear (Head (III)), respectively. It should be noted that both the retrieval heads were implanted without significant positioning error at the time of primary surgery, as far as the cup and stem orientation were concerned.

### 2.3. Raman Microprobe Spectroscopic Analyses

The monoclinic volume fraction, *V*_*m*_, and the residual (tensor trace) stresses, *σ*, of Head (I)~(III) were quantitatively measured by means of a Raman microprobe spectrometer (MS3504i, SOL instruments Ltd., Minsk, Republic of Belarus). The excitation source was a 488 nm Ar-ion laser (GLG3103, Showa Optronics Co., Ltd., Tokyo, Japan) yielding a power of approximately 35 mW on the sample surfaces. The confocal configuration of the probe adopted throughout the present experiments corresponded to a ×100 objective; numerical aperture, focal length, and pinhole diameter were fixed as 0.6, 7.6 mm, and 100 *μ*m, respectively. Individual spectra were typically collected in 5 seconds. All the spectra were acquired in backscattering geometry with a spectral resolution of ~1.5 cm^−1^ achieved by a 2400 grooves/mm grating. The recorded spectra of three successive measurements were averaged. The focal plane was eventually shifted from the articular surfaces down to 100 *μ*m insides in order to screen nondestructively the subsurface regions of the femoral heads. An* in plane* sampling of 2.5 *μ*m lateral steps was applied at each depth and a spectral map of 50 × 50 *μ*m^2^ dimensions was collected (for a total of 1323 spectra per each map). A total of 2205 different locations (= 441 points per map × 5 maps) were analyzed at each selected depth of the femoral heads.

Spectral deconvolution of all the recorded spectra into subbands was performed according to a mixed Gaussian/Lorentzian curve fit using computational software (Labspec 3, HORIBA Jobin-Yvon SAS, Lille, France). The band intensities and peak positions were calculated after spectral fitting, and the computations of *V*_*m*_ and *σ* were made according to (1) and (2), respectively [15–17]:

(1) Vm=0.5Im180+Im1902.2It150+0.5Im180+Im190,

(2) σ=ΔνΠ,

where *I*_*t*_ and *I*_*m*_ represent the intensities of the Raman bands from tetragonal and monoclinic phases in the 3Y-TZP, whose wavenumbers are identified by the subscript. Π and Δ*ν* represent the piezo-spectroscopic (PS) coefficient and the spectral shift of a Raman peak. Since Π values were previously reported as 1.33 and −1.55 cm^−1^ /GPa for tetragonal (250 cm^−1^) and monoclinic (460 cm^−1^) phase bands, respectively [17], Δ*ν* can be considered to be a direct measure of the residual stress trace stored within the volume probed by the laser beam for each spectral acquisition. In addition, the equilibrium stress (*σ*_eq_) for 3Y-TZP was calculated according to the following equation [17]:

(3) $$\begin{array}{c} \sigma_{eq}=\left(\;1-V_{m}\;\right)\sigma_{t}+V_{m}\sigma_{m}, \end{array}$$

where *σ*_*t*_ and *σ*_*m*_ represent the residual stress traces stored in the tetragonal and monoclinic phases, respectively. In the present study, the Raman peak positions obtained from the unused surfaces of Head (I) were taken as stress-free references in calculating the *σ* values according to (2)-(3). Therefore, the obtained stress values represent the relative changes of residual stress with respect to the unused implant device.

Moreover, the thickness of the transformed zone, *T*_*m*_, of Head (I) was determined through mathematical deconvolution, which is a procedure to clean up the experimental depth profile of *V*_*m*_ from the averaging effect of the Raman probe upon solving the following equation (for simplicity, we omit the full details of derivation of the formula and numerical methodologies here, since they have been explicitly given elsewhere [18]):

(4) Vmz0=∫z=0TmVmax⁡×exp⁡−z−z02/2R2dz+∫Tm+∞Vmax⁡×exp⁡−V1z−Tm+V2/1+V2×exp⁡−z−z02/2R2dz∫z=0Tmexp⁡−z−z02/2R2dz+∫Tm+∞exp⁡−z−z02/2R2dz,

where *z*_0_ and *z* are the focal position in the head surface and subsurface, respectively; *V*_max_ is the true maximum value of *V*_*m*_; *R* is the radius of the waist of the laser beam; and *V*_1_, *V*_2_, and *T*_*m*_ are the fitting parameters. The least-square curve fitting of the experimental data according to (4) was performed with the aid of commercially available software (Mathematica 7; Wolfram Research, IL).

## 3. Results and Discussion

### 3.1. Environmental Stability and LTD Kinetics of the 3Y-TZP Femoral Heads

**Figure 1:**
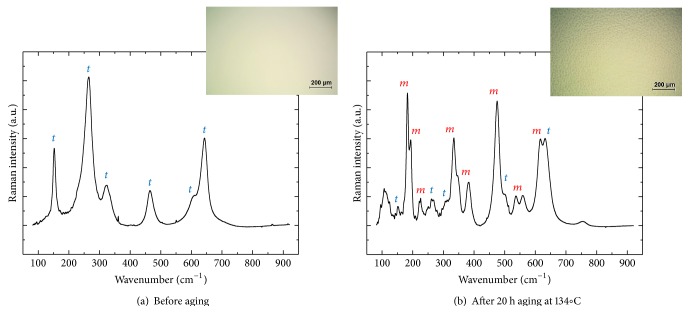
Average Raman spectra and optical microscopic images collected at the surfaces of Head (I) before (a) and after 20 h aging at 134°C (b).

**Figure 2:**
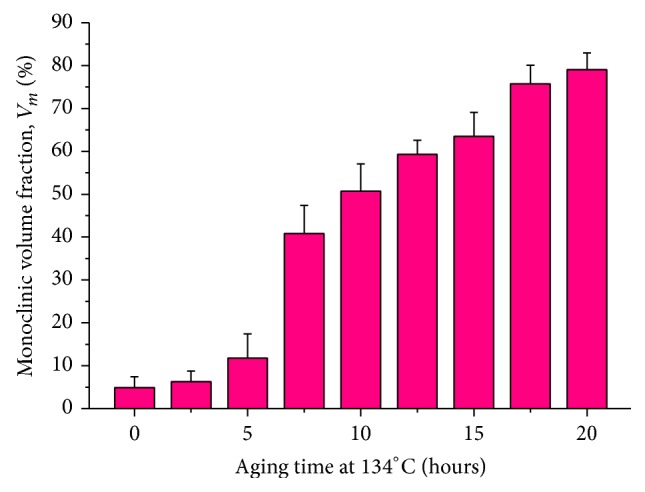
Raman spectroscopic data for the Head (I) surfaces showing the evolution of *t* → *m* phase transformation as a function of hydrothermal aging time* in vitro*.

Figures 1(a)-1(b) show representative Raman spectra collected from Head (I) before and after a 20 h aging period at 134°C. In the as-received state of Head (I), the observed Raman bands indicate predominant spectral contributions from tetragonal phase, while the bands from the monoclinic phase become the most preponderant after 20 h exposure to water vapor. Representative optical micrographs of the Head (I) surface are also given in the upper-right of each figure.  Figure 1(b) reveals the homogeneous formation of transformation-induced surface roughness with significant uplifts after autoclaving. The mean volume fractions of transformed 3Y-TZP (*V*_*m*_) were calculated from the fitted spectra of Head (I) surfaces using (1) and plotted as a function of* in vitro* aging time in Figure 2. The presence of a small fraction of monoclinic phase (*V*_*m*_ = 4.9%) was detected by Raman spectroscopy on the unused surface of Head (I), which was formed during the manufacturing process. The moisture-induced *t* → *m* transformation occurred slowly up to 5 hours aging, but it proceeded rapidly after 7.5 hours. This behavior can be interpreted according to a nucleation/growth model [3, 8, 19–21]. According to this model, the slow increase in monoclinic fraction observed at the initial stage of aging is due to the formation of monoclinic nuclei on the head surface. On the other hand, the sudden rise in monoclinic fraction after 7.5 hours aging represents the initiation of nuclei coalescence and, ultimately, the extension toward the bulk of the material.

In order to rationalize the LTD behavior of Head (I), the Mehl-Avrami-Johnson (MAJ) theory was applied. It has been previously reported that the kinetics of *t* → *m* transformation induced by aging can be expressed according to a modified MAJ equation [3, 8, 19–23]:

(5) $$\begin{array}{c} V_{m}=1-\left(\;1-{V_{m}}^{0}\;\right)\exp\left[\;-{\left(\;bt\;\right)}^{n}\;\right], \end{array}$$

where *V*_*m*_^0^ is the pristine monoclinic phase fraction in the material before the aging test, *b* is a parameter that represents the temperature dependence of the aging effect, *t* is the aging duration, and *n* is the time exponent, independent of temperature (generally referred to as the Avrami exponent). It should be noted that the *n* value reflects the proportion of nucleation and growth rates. Previous experimental data and simulations showed that the *n* exponent ranged between 0.5 and 4 [3, 20]. An *n* value close to 4 represents a preponderant contribution to the kinetics of transformation from the growth of preexisting monoclinic nuclei, while the nucleation rate is dominant at small *n* value [3, 8, 19–23]. The *n* exponent can be derived from the logarithmic form of (5) as follows:

(6) $$\begin{array}{c} \ln\left(\;\ln\left(\;\frac{1-{V_{m}}^{0}}{1-V_{m}}\;\right)\;\right)=n\ln\left(\;b\;\right)+n\ln\left(\;t\;\right). \end{array}$$

**Figure 3:**
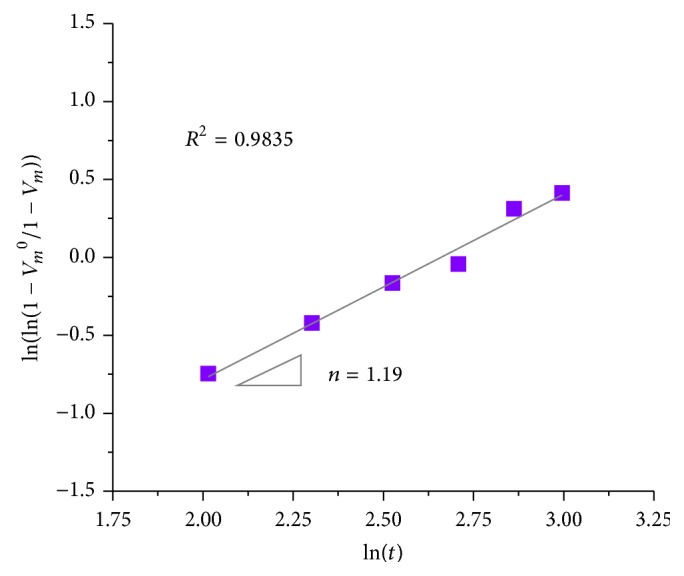
Plot of ln(ln(1 − *V*_*m*_^0^/1 − *V*_*m*_)) versus ln(*t*) for the determination of the Avrami exponent, *n*, for polymorphic transformation in Head (I). The exponent was obtained as *n* = 1.19 by retrieving the slope of the best regression line for the plot obtained by Raman spectroscopic measurement.

On the basis of the present experimental data, the obtained exponent value was *n* = 1.19 (*R* ^2^ = 0.9835) as calculated from the slope of the best regression line to a plot ln(ln(1 − *V*_*m*_^0^/1 − *V*_*m*_)) versus ln(*t*) (Figure 3). The *n* value between 1 and 2 corresponds to a nucleation and one-dimensional growth process [21]. A similar evaluation based on MAJ theory was made by other authors for a commercial femoral head made of zirconia-toughened alumina matrix composite (ZTA) with the trade name of BIOLOX* delta* (CeramTec AG, Plochingen, Germany), which is presently one of the most advanced ceramic femoral heads. The *n* values of BIOLOX* delta* were previously reported as 0.60~0.78 [8, 22, 23]. The studied Head (I) had a higher Avrami exponent as compared to BIOLOX* delta*, suggesting a faster growth rate of monoclinic nuclei in the LTD process. In other words, Prozyr head is expected to present faster kinetics of LTD during implantation.

### 3.2. Effects of Transformation-Induced Stresses on the 3Y-TZP Femoral Heads

**Figure 4:**
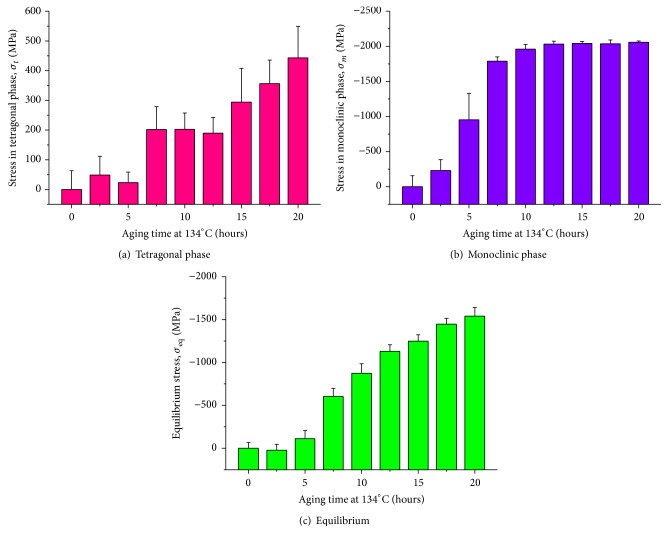
Raman spectroscopic data for the Head (I) surface showing the evolution of transformation-induced residual stresses in tetragonal (a) and monoclinic phase (b) as a function of the aging time. The equilibrium stress obtained according to (3) is also plotted in (c).

**Figure 5:**
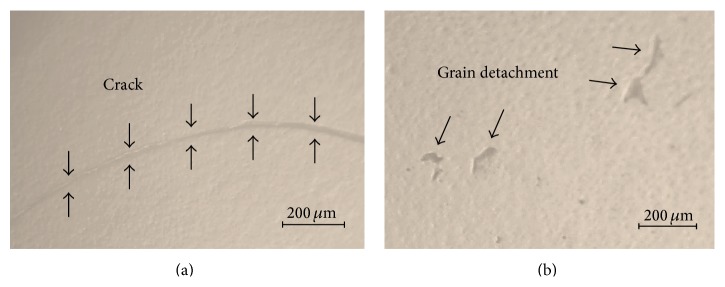
Optical micrographs showing surface crack propagation (a) and grain detachment (b) in Head (I) after 15 hours aging at 134°C.

Figures 4(a)-4(b) show the time-dependent plots of the average residual stresses stored in the tetragonal and monoclinic phases (*σ*_*t*_ and *σ*_*m*_, resp.) in Head (I) surfaces. As shown in these plots, polymorphic transformation generated tensile and compressive stresses in the tetragonal and monoclinic grains of Head (I), respectively. Residual stress accumulation clearly proceeded with increasing aging time. According to stress assessments made by Schubert and Frey [24], the maximum tensile stresses induced by destabilization of the tetragonal polymorph were approximately evaluated as 300~500 MPa, which is a stress range similar to that detected in this study. The stress magnitudes rapidly increased at 7.5 hours aging presumably due to initiation of the growth process for monoclinic nuclei. The monoclinic phase incorporated strong residual compressive stresses in the GPa order in Head (I) surfaces, whose values reached a saturation plateau after 10 hours. The equilibrium residual stress, *σ*_eq_, is also plotted in Figure 4(c), according to (3). Influenced by the quite strong compression generated in the monoclinic phase, the *σ*_eq_ value at the head surface increased toward the compression side with increasing aging time. However, extremely high stress gradients can be envisaged on the head surface, which mechanically destabilize the bearing surface. It should be noted that the LTD-induced tensile stresses are responsible for surface microcracking, and ultimately, for fracture of the prosthesis. Indeed, after 15 hours aging, crack formation and grain detachment were clearly observed by optical microscopy on the surfaces of Head (I) (cf. Figures 5(a)-5(b)), which incorporated a tensile stress >300 MPa in the tetragonal phase (cf.  Figure 4(a)). The largest tensile stresses could be found at grain junctions and edges where nucleation of transformation initiated, thus leading to the formation of cracks predominantly along grain boundaries [3].

**Figure 6:**
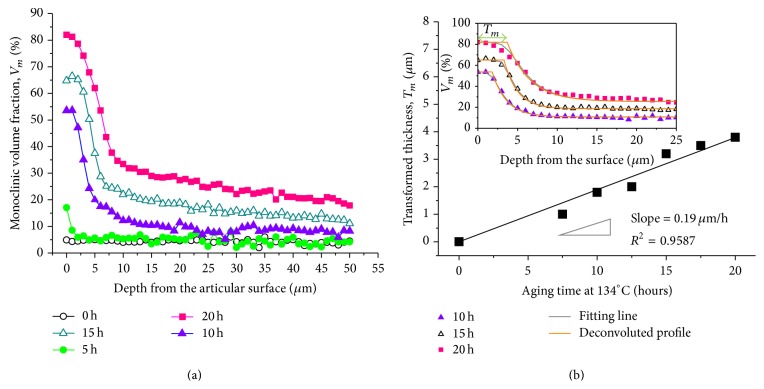
(a) Depth profiles of monoclinic phase fraction in Heads (I) after hydrothermal aging for different periods of time and (b) thickness of the transformed zone as a function of the aging time. The slope of the transformed thickness versus aging time indicates the rate of transformation propagation. The representative best-fitting curves of the experimental data (after 10, 15, and 20 hours aging) using (4) and deconvoluted monoclinic fraction profiles are also shown in the inset of (b).

Crack and grain-detachment formation, as observed in Figure 5, allows moist vapor to penetrate toward the head subsurface. The moisture can flow through grain boundary cracks and pores much faster than diffusion of hydroxyl ions into the lattice of individual grains [3]. Monoclinic phase fractions were plotted in Figure 6(a) as a function of in-depth distance along the subsurface in Head (I) after aging for increasing periods of time. All the analyzed samples exhibited the highest contents of phase transformation at their surface. These data prove that phase transformation actually proceeds from the free surface toward subsurface regions with increasing exposure time. The thickness of the transformed zone (*T*_*m*_) was plotted in Figure 6(b) as a function of the aging time, which was determined from the deconvoluted monoclinic profile, *V*_*m*_(*z*). According to the least-square method, the best-fitting curves determined using (4) with minimum deviations from the data points were also plotted in the inset of the figure, and the good agreement between theoretical and experimental plots was confirmed, indicating high degree of precision and reproducibility of the results. Figure 6(b) illustrates that the transformation process of Head (I) can propagate in a nearly linear way (slope = 0.19 *μ*m/h, *R* ^2^ = 0.9587), and the *T*_*m*_ value was 3.8 *μ*m after the 20 h exposure in vapor atmosphere. A simple linear time dependency in the aging kinetics of 3Y-TZP materials was also reported in the other recent studies [18, 25–28].

**Figure 7:**
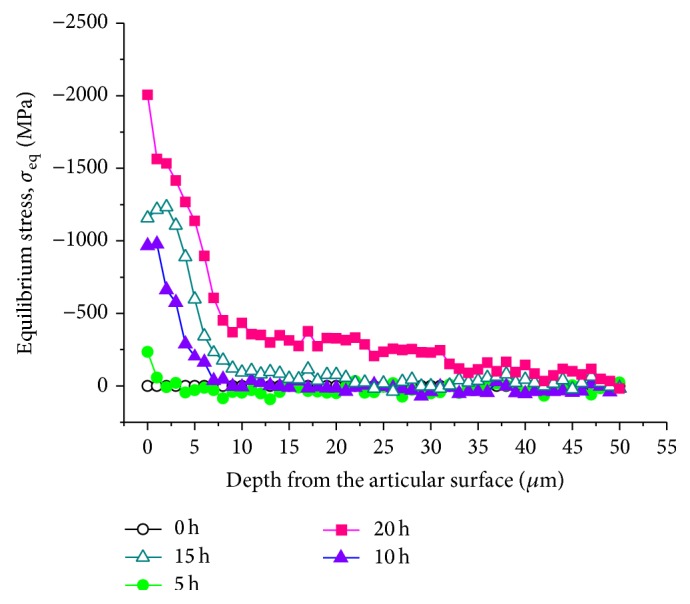
Depth profiles of equilibrium residual stress in Heads (I) after hydrothermal aging for different periods of time.

Since the tetragonal phase at the surface of the sample is more exposed (and unstable) as compared to the subsurface, the environmental stability is strongly dependent on the surrounding grains. Note that the gradual development of equilibrium residual stress is also observed within the first 50 *μ*m below the free surfaces due to the progress of polymorphic transformation (Figure 7).

### 3.3. Comparison of LTD Kinetics for* In Vitro* and* In Vivo* Environments

In the above Sections 3.1 and 3.2, the time-dependent LTD behavior of monolithic 3Y-TZP was simulated according to* in vitro* hydrothermal aging tests and nondestructively characterized by Raman microprobe spectroscopy. According to the rationale behind both ISO and ASTM standards [8–11], the* in vitro* aging time should theoretically be comparable to* in vivo* implantation periods. Nevertheless, when converting from* in vitro* to* in vivo* times according to activation energy arguments, one can find a clear discrepancy between ISO and ASTM criteria. It was reported that, for medical grade 3Y-TZP, one-hour exposure at 134°C under 2 bars water steam corresponds approximately to 4 years* in vivo* exposure according to ISO standard [8, 9]. On the other hand, ASTM F2345-03 defined that one-hour exposure at the above condition corresponds to 2 years* in vivo* [10, 11], which indeed represents a more severe condition for 3Y-TZP because of a shorter* in vivo* time for the same aging duration. The origin for this discrepancy lies in a difference in the activation energy assumed for the extrapolation to* in vivo* temperature. In this context,* in vitro* aging criteria should not be considered as rigorous and universally true in lifetime extrapolation* in vivo*, but just as a rough indication. However, despite the fact that the most frequently published studies to date refer to ISO standard, we shall show hereafter that this standard is not the most appropriate for 3Y-TZP assessments.

**Figure 8:**
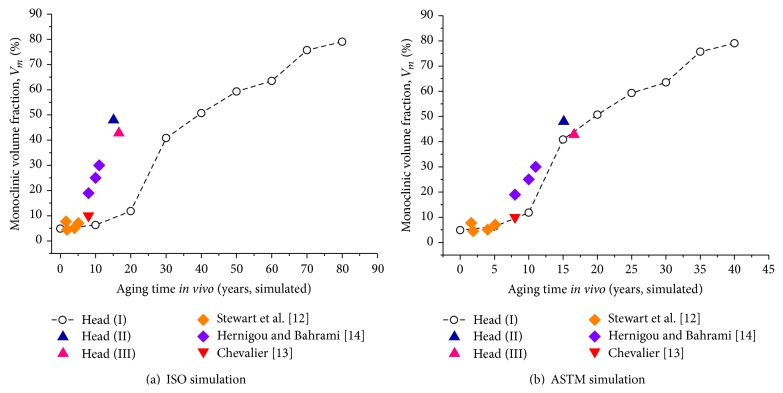
Monoclinic phase fractions on the surface of Head (I) are plotted as a function of* in-vivo* time simulated according to ISO (a) and ASTM criteria (b). Note that the comparisons of the transformation kinetics are made between* in-vitro* and* in-vivo* environment by coplotting the transformed contents on the surfaces of Heads (II) and (III) as well as monoclinic contents reported for Prozyr femoral head retrievals in [12–14].

In Figures 8(a) and 8(b), the monoclinic contents on the surface of Head (I) aged* in vitro* were compared with the 3Y-TZP head retrievals (Heads (II) and (III)) and other values obtained from the literature; plots are given as a function of* in vivo* times simulated according to ISO and ASTM recommendations, respectively. These plots can be helpful in understanding how realistic these standards are, and this provides the most practical estimation for the biological environment. In addition to our long-term retrievals (15.1 and 16.6 y), we also introduced in the plot results of Prozyr retrievals after short- and middle-term implantation (1.6~11 y), which had previously been reported in the literature [12–14]. Stewart et al. [12] reported 4.48~7.75% monoclinic contents between 1.6 and 4 years follow-up* in vivo*, while Chevalier [13] reported 10% transformation after 8 years* in vivo*. Hernigou and Bahrami [14] detected higher monoclinic contents at the levels of 19, 25, and 30% after 8, 10, and 11 years of* in vivo* implantation, respectively. It should be noted that the data plotted for Heads (II) and (III) represent the surface monoclinic fractions detected in their nonwear zones, predominantly including the influence of biological environment itself without any significant wear and load. According to Figure 7, the ASTM profile shows a much better agreement with the* in vivo* results as compared to the ISO profile. Accordingly, the ASTM recommendation appears as a more reasonable indication for predicting the rate of environmentally driven polymorphic transformation in medical grade 3Y-TZP. The obtained results also imply that, in Prozyr 3Y-TZP femoral head components, nucleation of monoclinic sites should be expected approximately within the first 10 years of implantation. Subsequently, an exponential growth of monoclinic nuclei occurs, leading to a markedly enhanced risk of surface roughening and fracture.

Here, we would like to highlight several potential limitations of this study for the readers' convenience. The first one certainly was the limited sampling of retrieved Prozyr femoral heads after various implantation periods (only a total of ten retrieval data collected after short- to long-term services were considered here, according to the currently available literature data [12–14]). Secondly, the femoral heads used in this study were the first generation of monolithic 3Y-TZP introduced clinically in 1989. Thus, the obtained LTD behaviors are not generalizable to other ZrO_2_-containing prostheses with different constituents, grain sizes, and manufacturing processes. In addition, it should be noted that, as seen in Figure 8(b), the middle-term retrievals (8~11 years) exhibited larger *V*_*m*_ values as compared to the ASTM simulation line. Since the literature data were obtained from the wear zones of each retrieval surface [12–14], an additional contribution of biomechanical origin should be considered on top of the hydrothermal effect on polymorphic transformation. Indeed, the wear zones in the longer-term retrievals of Head (II)-(III) had significantly higher contents of monoclinic polymorphs (detected as 66.8 and 54.5%, resp.) as compared to their respective nonwear zones. Thus, the interpretation of aging simulation for a long duration time should strictly be limited to predictions of the* in vivo* LTD in a nonwear zone. However, despite these limitations, we showed that the accelerated aging test could still be comparatively effective in investigating LTD processes and kinetics of 3Y-TZP femoral heads, giving reasonable predictions for the* in vivo* behavior, provided that the ASTM recommendation is adopted.

## Figures and Tables

**Table 1:** Summary of the clinical characteristics of the 3Y-TZP femoral heads investigated in this study.

Femoral head sample		Diameter	Maker	State	Follow-up	Cause of revision
Head (I)	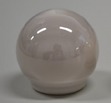	28 mm	Saint-Gobain Desmarquest	Unused or *in-vitro* aged at 134°C	—	—

Head (II)	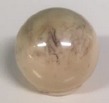	28 mm	Saint-Gobain Desmarquest	Retrieval	15.1 yrs	Infection

Head (III)	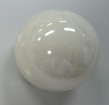	28 mm	Saint-Gobain Desmarquest	Retrieval	16.6 yrs	UHMWPE liner wear
